# LncRNAs in molluscan and mammalian stages of parasitic schistosomes are developmentally-regulated and coordinately expressed with protein-coding genes

**DOI:** 10.1080/15476286.2020.1729594

**Published:** 2020-03-04

**Authors:** Hyung Chul Kim, Ahmad M. Khalil, Emmitt R. Jolly

**Affiliations:** a Department of Biology, Case Western Reserve University, Cleveland, OH, USA; b Department of Genetics and Genome Sciences, Case Western Reserve University, Cleveland, OH, USA; c Case Comprehensive Cancer Center, Case Western Reserve University, Cleveland, OH, USA; d Center for Global Health and Disease, Case Western Reserve University, Cleveland, OH, USA

**Keywords:** Schistosoma, parasite, lncRNA, helminths, association prediction, differential expression

## Abstract

Despite the low level expression of some long noncoding RNAs (lncRNAs), the differential expression of specific lncRNAs plays important roles during the development of many organisms. Schistosomes, parasitic flatworms that are responsible for schistosomiasis, infects over 200 million people resulting in chronic disease and hundreds of thousands of deaths. Schistosomes have a complex life cycle that transitions between molluscan and mammalian hosts. In a molluscan snail host, the sporocyst stage develops over 5 weeks undergoing asexual reproduction to give rise to free-swimming and infectious cercariae that penetrate human skin and eventually mature into egg producing worms in mammals. The tight integration of the sporocyst to the snail host hepatopancreas hinders the -omics study in the molluscan stage, so the sporocyst transcriptome has only been examined for lncRNAs in immature *in vitro* samples. Here we analyzed the *in vivo* mature sporocyst transcriptome to identify 4,930 total lncRNAs between the molluscan and mammalian stages of the parasite. We further demonstrate that the lncRNAs are differentially expressed in a development-dependent manner. In addition, we constructed a co-expression correlation network between lncRNAs and protein-coding (PC) genes that was used to identify clusters of lncRNA transcripts with potential functional relevance. We also describe lncRNA–lncRNA and lncRNA–kinome correlations that identify lncRNAs with prospective roles in gene regulation. Finally, our results show clear differential expression patterns of lncRNAs in host-dependent development stages of *S. mansoni* and ascribe potential functional roles in development based on predicted intracellular interaction.

## Introduction

Regulation of gene expression is a key driver for cellular differentiation and development. Various types of noncoding RNAs (ncRNAs) have been shown to function as critical modulators of gene expression both at the transcriptional and post-transcriptional levels [1–5]. Among the various classes of noncoding RNAs, long noncoding RNAs (lncRNAs) have emerged as key players in gene regulation. These molecules, lncRNAs, are only currently defined as transcripts over 200 nucleotides in length with little to no protein-coding potential [6], but have diverse functional roles in gene regulation by interacting with DNA, RNA, proteins, and chromatin [7–12]. Furthermore, it has been demonstrated that substantial parts of the genome are transcribed, but only a small portion of the transcriptome comprises protein-coding (PC) genes [13–15]. Many of these transcribed regions correspond to numerous annotated but poorly characterized lncRNAs. Further, many lncRNAs have been shown to be expressed at low abundance compared to mRNAs, and expressed in a tissue-specific manner, emphasizing the role lncRNAs play in development [16,17]. Despite the important role of lncRNAs in key biological processes, the functional analysis of these lncRNAs is sometimes hindered by technical challenges in some biological systems, and so far only a small fraction of the identified lncRNAs have been analyzed for their functional role. Therefore, while it is important to identify these lncRNAs from various organisms, it is also critical to pinpoint lncRNAs with higher potential for functional roles to prioritize in functional analysis.

Schistosomes are parasitic flatworms that are the causative agent of schistosomiasis, a neglected tropical disease that affects over 240 million people worldwide. They have a complex life cycle that transitions between molluscan and mammalian hosts, in which they undergo asexual reproduction and sexual reproduction, respectively. A freshwater snail is infected with a short-lived and free-swimming miracidia. After invasion into the snail, the miracidia transforms into a mother sporocyst and undergo clonal expansion to produce daughter sporocysts [18]. The full maturation of the sporocyst occurs over 5 weeks and is marked by production of infectious cercariae. The establishment of the snail host *Biomphalaria glabrata* embryonic (Bge) cell line has allowed for *in vitro* culture of sporocysts, but these *in vitro* sporocysts are not able to mature sufficiently enough to produce cercariae [19]. Studies performed on *in vitro* sporocysts have used sporocysts that were 48-hour (48h) old or up to 20 days before sporocysts mature to produce cercariae [20,21]. As sporocysts develop, they integrate into the snail host hepatopancreas resulting in challenges to RNA sequencing analysis to distinguish snail transcripts from mature sporocyst transcripts. Thus, while lncRNA identification has been performed in *S. mansoni* [22–25], there has not been a lncRNA analysis in *in vivo* sporocysts.

In our study, we identified lncRNAs from *in vivo* sporocysts using a high-fidelity lncRNA identification tool optimized for use in non-model organisms. We filtered out snail host transcripts from sporocyst RNA-seq data to show that sporocysts not only express a unique set of lncRNAs, but also exhibit a distinctive lncRNA expression pattern for differential gene expression. We further constructed co-expression correlation networks for lncRNA–PC, lncRNA–kinome, and lncRNA–lncRNA interactions to highlight transcripts with promising functional potential.

## Results

### Separation of snail transcripts from sporocyst transcript allows in silico identification of lncRNA in *S. mansoni*

**Figure 1.**
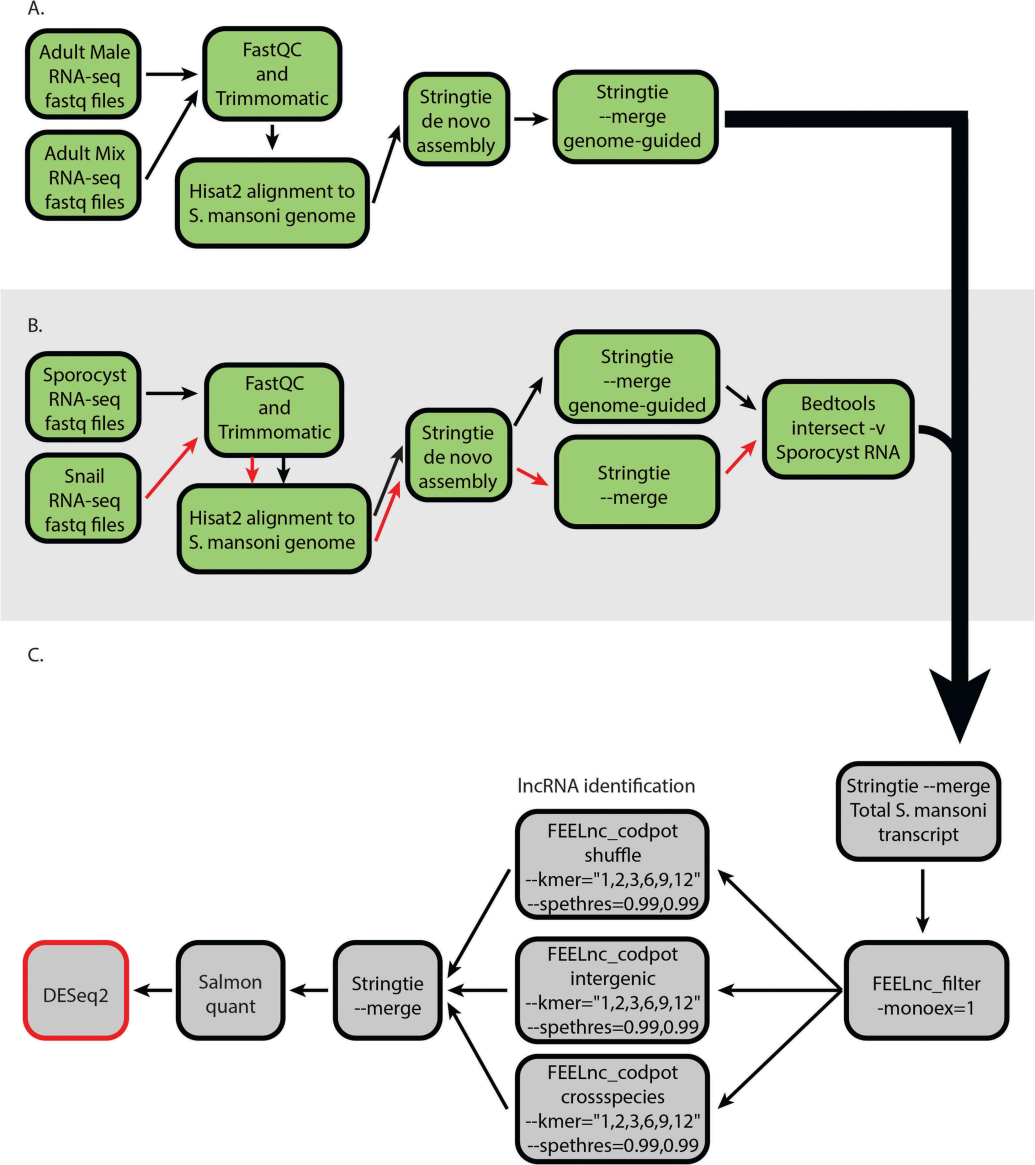
A computational pipeline was designed to identify lncRNAs from *S. mansoni* molluscan stage (asexual reproduction) and mammalian stage (sexual reproduction). (A) The general workflow for *de novo* assembly of the transcripts. The RNA-seq datasets were aligned to *S. mansoni* genome and assembled into transcripts. (B) The sporocyst and the snail RNA-seq datasets were aligned to *S. mansoni* genome and assembled into transcripts. The snail transcripts that align to *S. mansoni* genome were then filtered out from the sporocyst transcripts to obtain sporocyst-only transcripts. (C) lncRNA identification and differential expression analysis. The reconstructed transcripts from different developmental stages were merged together, and the coding potential was calculated for each transcript. The identified lncRNAs were used as the reference for quantification of transcripts from RNA-seq datasets. The quantified transcripts were then analyzed for differential gene expression in different developmental stages.

Sporocysts infect the molluscan hosts by invading the snail hepatopancreas. As such, the complete separation of *in vivo* sporocyst from snail tissue is difficult. Consequently, we used a computational pipeline to filter the snail transcripts from sporocyst RNA-seq datasets (Fig. 1). We aligned the sporocyst datasets and uninfected snail datasets to *S. mansoni* genome, and the snail transcripts that aligned to the genome were then filtered out from sporocyst transcripts to obtain sporocyst-only transcripts. The mixed adult (made up of male and female worms) and male-only adult samples were also aligned to the genome and assembled into transcripts.

The lncRNA identification process was performed with FEELnc, a high-performing lncRNA identification program optimized to use in non-model organisms [26]. As lncRNA identification in other organisms has shown that some lncRNAs can be monoexonic, the filtering process retained any intergenic and antisense monoexonic transcripts [27–29]. To increase the sensitivity and specificity of the process, we used a relaxed ORF definition, multi k-mer frequencies, and increased the specificity threshold of mRNA and lncRNA [26]. Additionally, we maximized the lncRNA identification sensitivity by utilizing all three available strategies to model noncoding transcripts for the machine learning algorithm, then merged them together. The default FEELnc codpot module calculates an optimal threshold and assigns all transcripts as either lncRNA or mRNA based on the optimal threshold. To significantly decrease false positive rates, we increased the specificity thresholds for segregating transcripts into lncRNA or mRNA to 0.99 each as a part of the coding potential calculation process (Fig. 1C). This results in three categories of transcripts, lncRNAs, mRNAs and transcripts of unknown coding potential (TUCp). We excluded TUCp from further analysis. This approach resulted in lncRNAs with high confidence level values. The identified lncRNAs were then quantified from the RNA-seq datasets using the mapping-based mode of Salmon [30]. This pipeline identified novel lncRNAs along with previously identified lncRNAs. So, any transcripts with 99% sequence identity match with a previously identified lncRNA were given the previously assigned transcript ID. Furthermore, the lncRNA sets contained 27 transcripts that each aligned to known but uncharacterized schistosome Smp gene IDs. However, 14 of these transcripts were identified as noncoding pseudogenes and 13 of these transcripts are annotated to code for small peptides under 100 amino acids in length. Therefore, the transcript ID was reassigned to a lncRNA nomenclature, but we retained the Smp gene ID for the annotation as these transcripts have potential to be lncRNAs that code for small peptides. In the end, we identified 4930 lncRNA transcripts from 3687 genes (1.34 average isoform per lncRNA). Of the identified transcripts, 969 were on chromosome 1, 693 on chromosome 2, 591 on chromosome 3, 457 on chromosome 4, 470 on chromosome 5, 279 on chromosome 6, 193 on chromosome 7, and 1150 on the ZW chromosomes (S Table 1). Additionally, 62 transcripts aligned to W chromosome-specific contigs, another 62 transcripts aligned to contigs with unknown chromosomal origin, and 4 transcripts aligned to the mitochondria. The principal component analysis of the identified lncRNAs and the PC transcripts showed that the mixed adults, male adults, and sporocysts display distinct expression profiles (Supplementary Fig. 1). Therefore, we next investigated the extent of the differential expression of lncRNAs in these life stages.

### Sporocysts express unique sets of lncRNAs

**Figure 2.**
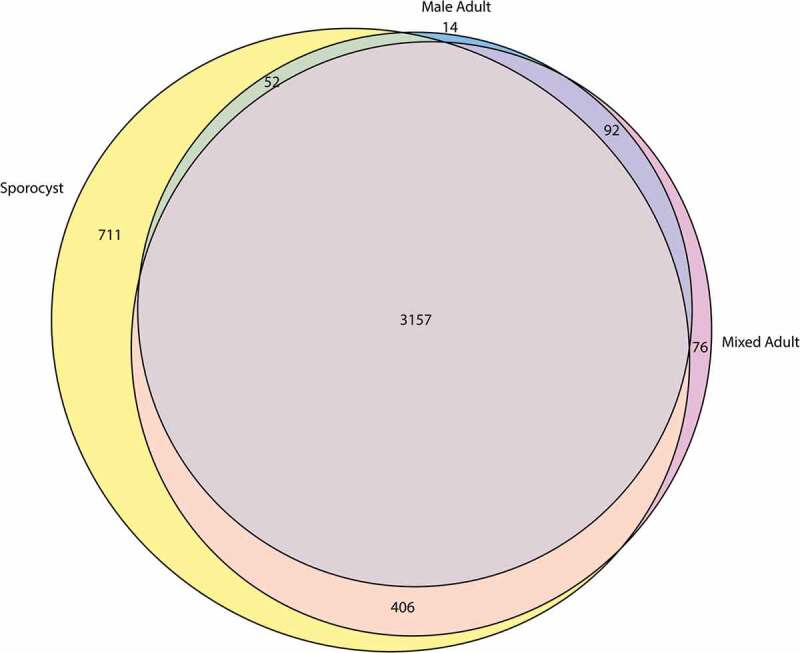
Euler diagram of the newly identified lncRNAs that are differentially expressed *S. mansoni*. Only the transcripts with average TPM ≥ 1 from triplicate datasets were included for this analysis.

The complex schistosome life cycle transitions between molluscan and mammalian stages that provide vastly different environments for the parasite. As each life stage manifests unique gene expression profile, we speculated that the sporocyst and adult worms that infect different hosts should express highly differential pattern of lncRNA. Among the 4930 identified lncRNA transcripts, we found that 3157 (64.0%) of the transcripts were expressed by mixed adults, male adults, and sporocysts. The mixed adults and male adults shared 92 (1.9%) transcripts, while sporocysts shared 406 (8.2%) and 52 (1.1%) of the transcripts with mixed adults and male adults, respectively. The three life stages also expressed unique transcripts with the mixed adults having 76 (1.5%) unique transcripts, male adults having 14 (0.3%) unique transcripts, and sporocysts having 711 (14.4%) unique transcripts (Fig. 2). These predictions suggest that each life cycle has a unique lncRNA expression profile and sporocysts express a large number of unique lncRNAs compared to adults.

Previously, lncRNAs were identified in other life stages of *S. mansoni*, but these methods utilized 48h *in vitro* sporocyst instead of mature *in vivo* sporocyst [22,25]. As such, we combined the previously identified lncRNA set with the lncRNAs identified with the *in vivo* sporocyst to estimate the total lncRNA expression profile in sporocyst and adults. Of the combined 21,512 total lncRNA transcripts, the mixed adults, male adults, and sporocysts shared 7397 (34.4%) transcripts. The sporocyst had 1730 (8.0%) and 358 (1.7%) transcripts in common with mixed adults and male adults, respectively, and the mixed adults and male adults shared 355 (1.7%) transcripts. Each life stage also expressed an exclusive set of lncRNAs, with 414 (1.9%) transcripts in mixed adults, 180 (0.8%) transcripts in male adults, and 4779 (22.2%) transcripts in sporocyst (Supplementary Fig. 2). As 711 transcripts from these 4779 sporocyst-specific transcripts were novel lncRNA transcripts, only 14.9% of the sporocyst-specific transcripts represent the novel lncRNAs identified from *in vivo* sporocyst. Nevertheless, with the total identified lncRNAs, the sporocyst continues to exhibit a larger set of lncRNAs compared to the adult stages.

**Figure 3.**
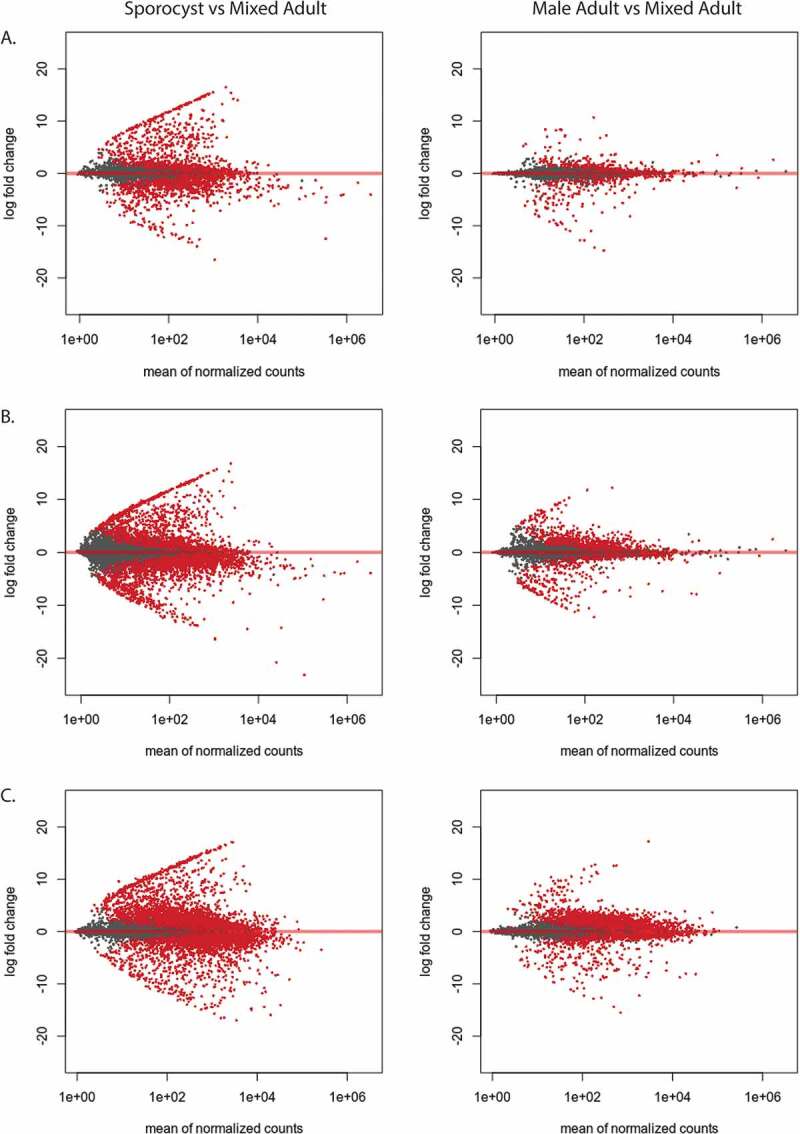
lncRNAs from adult and sporocyst are highly differentially expressed. Red dots indicate transcripts that are significantly differentially expressed (p < 0.01). (A) MA-plot of the newly identified lncRNA transcripts from sporocyst and male adults were compared to mixed adult transcripts. (B) MA-plot of the total lncRNA transcripts from sporocyst and male adults were compared to mixed adult transcripts. (C) MA-plot of protein-coding transcripts from sporocyst and male adults were compared to mixed adult transcripts.

Next, we performed differential gene expression analysis to assess how the unique and shared transcripts are expressed between the different life stages. The log2 fold change (log2FC) of the lncRNAs in sporocyst and male adult were compared against mixed adults. From the newly identified set of lncRNAs, the sporocyst exhibited 1281 (26.0%) upregulated lncRNA transcripts and 1193 (24.2%) downregulated lncRNAs (p < 0.01). In male adults, 441 (8.9%) of the lncRNAs were significantly upregulated, while 426 (8.6%) lncRNAs were significantly downregulated (p < 0.01) (Fig. 3A). We then examined the differential gene expression with the total lncRNAs that combined the newly identified set of lncRNAs with previously identified lncRNAs. In sporocyst, 2589 (12.0%) of the lncRNAs were significantly upregulated, while 2934 (13.6%) lncRNAs were significantly downregulated (p < 0.01). In male adults, 1093 (5.1%) of the lncRNAs were significantly upregulated, while 991 (4.6%) lncRNAs were significantly downregulated (p < 0.01) (Fig. 3B).

We then examined the differential expression of PC transcripts between sporocyst and male adults compared to mixed adults. Out of 14,528 PC transcripts, sporocysts had 5001 (34.4%) PC transcripts upregulated, while 4495 (30.9%) transcripts were downregulated. Male adults had 3,199 (22.0%) transcripts upregulated, while 3826 (26.3%) transcripts were downregulated (Fig. 3C). The highly differential nature of the lncRNAs in sporocyst and male adult compared to the expression of PC transcript suggests that the dynamic expression of specific lncRNAs is likely contributing to parasite development.

The sporocyst is continuously producing cercariae through its lifetime. Therefore, a mature sporocyst contains cercariae of various developmental stages. We analyzed the *in vivo* sporocyst-specific lncRNAs to observe whether these transcripts are expressed in cercariae. We selected the 711 *in vivo* sporocyst-specific novel lncRNAs and assessed cercariae RNA-seq datasets correlating to these transcripts. In cercariae, 150 (21.1%) of the sporocyst-specific novel lncRNAs were upregulated, while 326 (45.9%) of the novel lncRNAs were downregulated compared to sporocysts (p < 0.01) (Supplementary Fig. 3A). Comparing the PC transcripts between the two life stages, 3242 PC transcripts (22.3%) were upregulated in cercariae, while 3450 (23.7%) were downregulated (Supplementary Fig 3B). The high proportion of *in vivo* sporocyst-specific lncRNAs again shows the dynamic changes of lncRNA expression in the schistosome life cycle.

### qRT-PCR assays confirm lncRNA differential expression in different developmental stages

**Figure 4.**
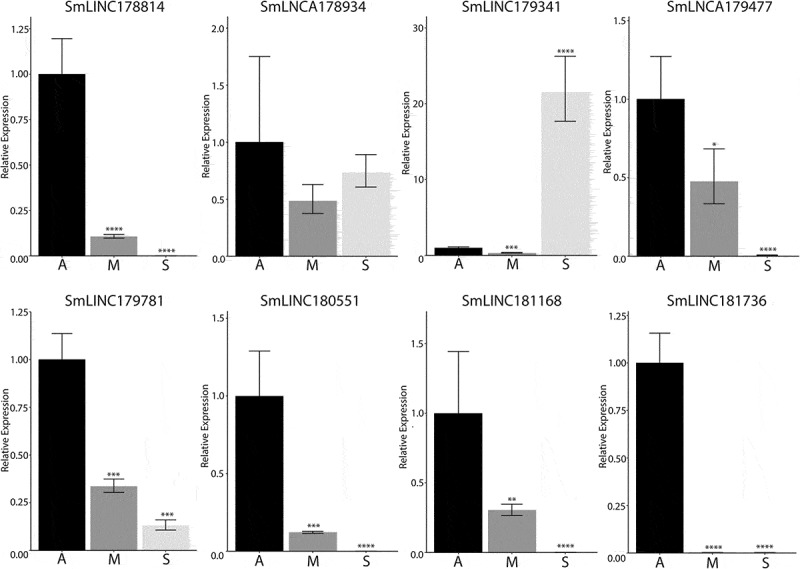
RT-qPCR to confirm the differential expression of identified lncRNAs. Eight lncRNAs with predicted differential expression patterns between sporocysts and adults were chosen for expression confirmation in mixed adults (A), male adults (M), and sporocyst (S). Mixed adult lncRNA expression was given the value of 1 and the relative lncRNA expression of sporocyst and male adults were compared against the mixed adults (*p-value ≤0.05, **p-value ≤0.01, ***p-value ≤0.001, ****p-value ≤0.0001). SmLINC178814, SmLINC179341, SmLINC179781, and SmLINC180551 are monoexonic transcripts. The endogenous cyclophilin (Smp_054330) was used as the reference gene for normalization between the life stages.

To validate our pipeline of identification and differential expression of lncRNAs, eight lncRNAs were selected for qRT-PCR analysis. These lncRNAs were selected on the basis that they are differentially expressed between adults and sporocyst for clearer verification of gene expression. Additionally, four of the seven selected lncRNAs are monoexonic to further verify the expression of monoexonic lncRNA transcripts. Of the eight lncRNAs, we detected expression from all eight lncRNA transcripts, and six followed the trend of the observed differential expression pattern between sporocysts and adults (Fig. 4). These observations confirm that these predicted lncRNAs are expressed and exhibit development-dependent patterns in *S. mansoni*.

### Co-expression networks identify potential hub lncRNAs

While there are multiple strategies for functional analysis of lncRNAs, the dearth of available molecular tools thwarts the potential functional assays in *S. mansoni*. Knockdown or knockout strategies like RNAi [31] and CRISPR [32,33] have been employed for the functional analysis. However, the lack of specific targeting methods and the lack of a viable cell line prevents an efficient functional analysis in *S. mansoni*, since lncRNAs are expressed in a more tissue-specific pattern than mRNA [16,17]. Notwithstanding, the co-expression-based correlation network has been shown to be able to predict the expression pattern of other genes in tissue-specific manner [34]. Therefore, to explore potential functional analysis in the future, we applied a co-expression-based correlation network to identify potential lncRNAs that may play regulatory roles on PC genes.

We selected the newly identified lncRNAs and PC transcripts that have TPM > 1, and higher level of differential expression from mixed adult samples (p < 0.01). The Pearson correlation analysis was subsequently performed on the resultant 1790 lncRNAs and 8371 PC transcripts and the relationships with *r* > 0.99 and *r* < −0.99 were retained. The resulting network showed that 1317 lncRNAs and 4407 PC transcripts exhibited 566,818 positive and 43,076 negative relationships (Supplementary Fig. 4A). Next, we investigated how many of these correlations are between lncRNAs and kinases to identify potential hub lncRNAs that may have a functional role in signaling pathways. Of the PC transcripts used in the previous analysis, we identified 479 kinases and 55 kinase-associated transcripts, for which we performed the co-expression analysis. At *r* > 0.99 and *r* < −0.99, a network between 1045 lncRNAs and 173 kinases was mapped to retrieve 19,962 positive and 948 negative relationships between lncRNAs and kinases (Supplementary Fig. 4B). Previous lncRNA–RNA interaction predictions have considered all RNA–RNA relationships, including lncRNA–mRNA and lncRNA–lncRNA interaction, so we further examined lncRNA–lncRNA interaction correlation maps to predict how lncRNAs may have regulatory functions with each other [35]. The interaction network between the selected 1293 lncRNAs predicted 237,232 positive and 116 negative relationships (Supplementary Fig. 4C).

**Figure 5.**
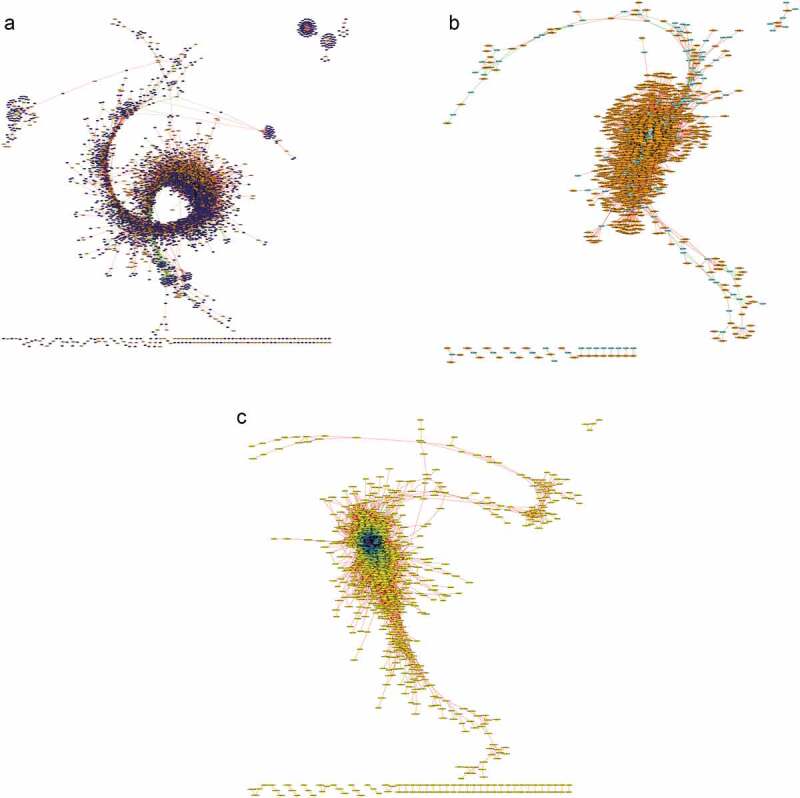
Correlation network of total lncRNAs based on co-expression. Expression levels of the lncRNA and PC transcripts were used to build a correlation network. (A) The lncRNA and PC transcripts with TPM >1 that are significantly differentially expressed between developmental stages (p < 0.01) were chosen for co-expression analysis at Pearson correlation value of *r* > 0.99 and *r* < -0.99. Between 2610 lncRNA (orange nodes) and 5255 PC transcripts (blue nodes), we found 943,027 positive correlation interactions (red edges) and 84,618 negative interactions (green edges). (B) Kinome transcripts were isolated from the PC transcripts to build a correlation network between lncRNAs and kinases for potential functional relevance of lncRNAs in signalling pathways. With 479 kinases and 55 kinase-associated transcripts (cyan nodes), we found 34,383 positive correlation interactions and 2020 negative interactions with lncRNAs. (C) Correlation network between lncRNAs was constructed between the 2610 lncRNAs to map 546,192 positive correlation interactions and 461 negative interactions. Node color ranges from yellow to purple for lncRNAs with fewer interactions to many interactions, respectively.

We then performed the co-expression correlation analysis with the combined list of previously identified lncRNAs and the newly identified lncRNAs. The Pearson correlation analysis was performed on 3565 lncRNAs and the 8371 PC transcripts used in the previous analysis. The lncRNA–PC co-expression correlation showed a network between 2610 lncRNAs and 5255 PC transcripts with 943,027 positive and 84,618 negative relationships (Fig. 5A). The list of kinase-associated transcripts was extracted from PC transcripts to reveal that 1973 lncRNAs and 213 kinases have 34,383 positive and 2020 negative relationships (Fig. 5B). The lncRNA–lncRNA network from the 3565 significantly differentiated lncRNAs showed 546,192 positive and 461 negative relationships (Fig. 5C). These co-expression correlation maps provide the hub lncRNAs with promising potential functional roles and identify potential clusters of genes that may be regulated by lncRNAs.

## Discussion

In this study, we designed a pipeline to utilize *in vivo* sporocysts in transcriptomic studies in order to identify lncRNA expression. We identified 4930 lncRNAs from sporocysts and adults, and confirmed their expression to validate the computational pipeline. We then provided data that sporocysts and adults express unique lncRNAs that are only expressed at specific developmental stages, and that overall lncRNAs are differentially expressed to give distinct expression pattern in each life stage. Finally, we modeled predicted interaction network between the identified lncRNAs and the PC genes, specifically focusing on kinome, using the co-expression correlation. Further, we explored potential lncRNA–lncRNA interactions via co-expression correlation to identify lncRNAs with high potential for functional roles for prioritizing future studies. Our findings support that lncRNAs expression is developmentally regulated as often observed in other eukaryotes [5,27,36,37]. The number of noncoding RNAs in an organism has been correlated with developmental complexity, potentially indicating a key role of lncRNAs in both cellular differentiation and identity [38,39]. We found that the schistosomes with extreme morphological changes throughout the complex life cycle fit in between *C. elegans* and vertebrates in terms of the proportion of noncoding regions in the genome. Moreover, our *in vivo* sporocyst filtering method also provides a potential mechanism to perform other omics studies in mature sporocysts over *in vitro* samples.

Sporocysts and adult schistosomes not only have a vast difference in morphology but also in many biological processes, such as in reproduction and in host immune evasion. Consequently, we expected to see noticeable differences in gene expression between these stages as has been observed in PC transcriptomic studies. We demonstrated that sporocysts and adult worms maintain distinct expression pattern in both lncRNAs and PC genes, and that sporocysts express a large set of unique lncRNAs. Our qPCR validation not only showed similar expression patterns of some lncRNAs between sporocysts and adult worms, but also showed differences in predicted patterns in mixed adults and male adults. The low abundance of lncRNAs can affect high cell-to-cell heterogeneity, which can explain the discrepancy of lncRNA levels between two adult populations [40]. However, these differences between adult samples also indicate that lncRNAs may be differentially expressed between male and female adults. As lncRNAs expression can be tissue-specific, the lncRNA expression pattern between the adult gonads may increase our understanding of schistosome reproduction and the regulation of asexual and sexual reproduction between sporocysts and adult worms.

As mentioned, sporocysts exhibit a unique set of lncRNAs that were not expressed in adults. Since a sporocyst contains various stages of developing cercariae, we further investigated whether these sporocyst-specific lncRNAs were conserved in mature cercariae. We found that of the 711 sporocyst-specific lncRNAs, 150 (21.1%) were upregulated and 326 (45.9%) were downregulated in cercariae, showing that 235 (33.0%) of the sporocyst-specific lncRNAs do not change the expression level significantly in cercariae. This 33% of the static lncRNAs suggest that some of these transcripts were identified from more advanced stages of the developing cercariae inside sporocysts, and that some lncRNAs may be pre-packaged from sporocysts and passed on to the cercariae. Moreover, the other 67% of the dynamic lncRNAs suggest that some lncRNAs may function in the maintenance of sporocyst and the development of the cercariae, and that the lncRNA expression may change once the cercariae exit the snail host and get exposed to external environmental signals. The comparison of sporocyst-specific lncRANAs in cercariae consequently highlights that lncRNAs are regulated throughout development and may serve a crucial role in the transition from one life stage to the next stage.

The co-expression correlation network was proposed as a method to identify lncRNAs with high functional potential by examining the hub lncRNAs or clusters of genes correlated to lncRNAs. These correlation clusters can act as a guide to pinpoint lncRNAs that can be prioritized in functional analysis. Gene expression correlation analysis has been shown to be effective in predicting expression patterns of other genes at tissue-specific level [34]. The specificity of this analysis would require single-cell RNA-seq and a gene expression atlas to provide enough tissue-dependent gene expression mapping to predict accurate lncRNA expression pattern. However, once applied in *S. mansoni*, this tool may provide a clearer view of lncRNA functional analysis.

The diverse potential roles for lncRNA require broader approaches for functional analysis. One of the criteria used for the coding potential calculation was the ORF percentage. Recent findings suggest that some lncRNAs undergo translation to produce small peptides from these short ORFs and that they may have regulatory functions [41,42]. Of the identified novel lncRNAs, we found 27 transcripts that aligned to schistosome Smp gene IDs that have already been annotated. However, these transcripts were either noncoding pseudogenes or protein-coding genes with low ORF percentages encoding small peptides under 100 amino acids in length. While none of these 27 genes have been experimentally verified, this alignment of lncRNAs to the annotated protein-coding genes underscores the small peptide translation form lncRNAs as a potential mechanism of lncRNA function in schistosomes. Additionally, m^6^A modification of RNA can affect RNA–protein interaction in lncRNAs, and promote translation in mRNA [43–45]. Thus, epitranscriptomic approaches may provide novel lncRNA mechanisms in *S. mansoni* and help in verifying small peptide translation from lncRNAs. Further, RNA has been identified as a key molecule for nucleation of some condensate formation [46]. Thus, the analysis of lncRNAs in biomolecular condensate may reveal the role of lncRNAs in many biological processes. We have identified developmentally regulated novel lncRNAs and constructed network of hub lncRNAs and gene clusters with potential functional roles. Therefore, the functional analysis for these lncRNAs should be prioritized to understand the biology of the parasite to investigate future therapeutic targets.

## Methods

### Animals and parasites

*B. glabrata* snails infected with *Schistosoma mansoni* (NMRI strain) were obtained from Biomedical Research Institute (BRI; Rockville, MD). Sporocysts-infected hepatopancreases were excised from infected snails and the snail hepatopancreas were collected from uninfected snails maintained in the laboratory at 5 weeks post-infection. Mixed male/female adult RNA and male adult RNA were also received from Biomedical Research Institute (BRI; Rockville, MD).

### RNA extraction

RNA was extracted from sporocysts and snail hepatopancreases as directed using the Direct-zol RNA Miniprep Kit (Zymo Research, Irvine, CA). RNA concentration and quality were assessed on a Nanodrop 8000 spectrophotometer (Thermo Scientific, Waltham, MA).

### Genomic and transcriptomic data analysis

The *S. mansoni* genome sequence and annotation were downloaded from WormBase ParaSite [47,48]. The most recent version (release 14) was used for the analysis presented here.

Fifteen RNA-Seq datasets were used for this study: (1) three sets of in-house sporocyst dataset composed of ~100 million raw paired-end reads from 5-week old sporocyst-infected snail hepatopancreas, (2) three sets of in-house male adult-only dataset composed of ~100 million raw paired-end reads from male-separated adults, (3) three sets of in-house mixed adult dataset composed of ~100 million raw paired-end reads from male and female adults, and (4) three sets of in-house snail dataset composed of ~100 million raw paired-end reads from uninfected *B. glabrata* snail, (4) cercaria RNA-seq datasets downloaded from the European Nucleotide Archive, accession numbers ERR022872, ERR022877, and ERR022878. The datasets were ran through FastQC for quality check [49] and the adapters were trimmed using Trimmomatics [50]. The datasets were then mapped to the *S. mansoni* genome using HISAT2 [51] followed by the use of BEDtools to remove snail transcripts aligning to *S. mansoni* genome from sporocyst dataset [52]. The filtered sporocyst datasets, adult-male datasets, and adult-mixed datasets were merged into a single .gtf file using the merge function of StringTie [53]. The cercaria RNA-seq datasets were quantified novel lncRNAs and PC transcripts using Salmon [30].

### Identification of long noncoding RNAs using next-generation RNA sequencing (RNA-seq)

The merged assembly of the transcripts was processed through FEELnc for lncRNA identification and classification [26]. FEELnc_filter_ was used to remove transcripts that overlap with exons from PC genes of the reference annotation while retaining single exon transcripts that are intergenic or antisense to PC genes. Next, FELLnc_codpot_ was used to calculate coding potential based on ORF length, nucleotide sequence bias, and transcript length to separate lncRNAs from mRNAs. The coding potential calculation was performed with optimized parameters with relaxed ORF definition, high specificity threshold, high sensitivity threshold, and multi k-mer frequencies. We calculated coding potential based on all three lncRNA sequence simulations – shuffle, intergenic, and cross-species. For cross-species mode, we used the sequences from previously identified *S. mansoni* lncRNA sequences [25]. The identified lncRNAs were subsequently processed through FEELnc_classifier_ to be classified based on their positions relative to the nearest PC gene. The transcripts with unknown strandedness relative to a proximal PC were blasted to the genome and were assigned strandedness based on its partner PC [54–56].

### RT-qPCR assays

Primers specific to SmLINC181168 (forward primer oHK157: 5ʹ- TCGCTATCATTCTCATCCTCAATATC-3ʹ, reverse primer oHK158: 5ʹ- TCATTCAAGCTTACTAGTATCCTCATC-3ʹ), SmLINC181736 (forward primer oHK159: 5ʹ- TCATAGAACGTCAACGTGAGTAAA-3ʹ, reverse primer oHK160: 5ʹ- GGAGTGTGTGCTGATGATGT-3ʹ), SmLINC179341 (forward primer oHK185: 5ʹ- CAACGGTCAAGGTTAACTGATAGA-3ʹ, reverse primer oHK186: 5ʹ- TCCAGTATGCGTATTCACGTAAG-3ʹ), SmLINC178814 (forward primer oHK191: 5ʹ- AACACGGTCTACCACACTATTC-3ʹ, reverse primer oHK192: 5ʹ- ACTGAACTTCACTTGGAACAAA-3ʹ), SmLINC180551 (forward primer oHK193: 5ʹ- GTGTGACTGCGTGAAATGTAAG-3ʹ, reverse primer oHK194: 5ʹ- AACACGGTCTACCACACTATTC −3ʹ),), SmLINC179781 (forward primer oHK195: 5ʹ- AAAGAAGCGGTTCGAGTACAA-3ʹ, reverse primer oHK196: 5ʹ- CATCCACTCGACCACAAGATATAA-3ʹ), SmLNCA178934 (forward primer oHK197: 5ʹ- AACGTGATATCGGTTAGCATCTC-3ʹ, reverse primer oHK198: 5ʹ- CATCCACTCGACCACAAGATATAA-3ʹ), and SmLNCA179477 (forward primer oHK199: 5ʹ- AACTGTTGGCGTTGATGATATTG-3ʹ, reverse primer oHK200: 5ʹ- ACATCAGTAGCACACACCATATT-3ʹ) were designed using the PrimerQuest web tool. Endogenous cyclophilin gene was used as the reference gene (forward primer oMV234: 5ʹ- AAATGGGTGGATTCAAGGTG-3ʹ, reverse primer oMV235: 5ʹ-TGTGACGTCCAGAATTAGCC-3ʹ). qRT-PCR reactions were carried out using Power SYBR Green RNA-to-C_T_ 1-Step Kit (Applied Biosystems, Foster City, CA). The qPCR was performed on two Q-qPCR machines (QuantaBio, Beverly, MA). The no-RT reactions were set up for each life stage RNA to determine DNA contamination. All experiments utilized the same RNA samples used for RNA-sequencing. Furthermore, the experiments were performed in triplicate and ΔΔCT method was used to analyze the transcript expression levels relative to the mixed adult stage.

### Statistical analyses

The transcripts were quantified using the mapping-mode of Salmon with the identified lncRNAs as reference transcripts [30]. Statistical analyses were performed with R environment [v3.4.4: 57] with DESeq2 [58], readr [59], and tximport [60] libraries loaded. The log2 fold change shrinkage was performed with the apeglm package [61]. P values used for all analyses were adjusted for multiple testing.

### Co-expression analysis

We selected lncRNAs and PC genes that have TPM > 1 and were significantly differentially expressed between mixed adult vs. sporocyst and mixed adult vs. male adult (p < 0.01) for correlation-based co-expression analysis (Supplementary data 8). We used R to get Pearson correlation of lncRNA–PC gene pair from the raw count of lncRNAs and PCs from RNA-seq datasets [57]. Gene pairs with *r* > 0.99 and *r* < −0.99 were assigned as positive and negative relationships, respectively. The correlation results were assembled into simple interaction formats (.sif) and processed in Cytoscape program to construct the co-expression map [62]. Next, 479 kinases and 55 kinase-associated transcripts were selected from the PC genes to a separate kinome dataset. The lncRNA–kinome co-expression dataset was obtained from the correlation between the selected lncRNAs and the kinome dataset. Subsequently, the lncRNAs were then compared against each other to obtain a lncRNA–lncRNA co-expression dataset and mapped using Cytoscape.

## Supplementary Material

Supplemental Material

## Data Availability

The RNA-seq datasets generated for this study are available at NCBI’s Sequence Read Archive under Accession number PRJNA602007.
