# Supplementary figures and images for: LncRNAs in molluscan and mammalian stages of parasitic schistosomes are developmentally-regulated and coordinately expressed with protein-coding genes
Source: RNA Biol. 2020 Mar 4;17(6):805–15. doi: 10.1080/15476286.2020.1729594 (PMC7549628; doi:10.1080/15476286.2020.1729594)

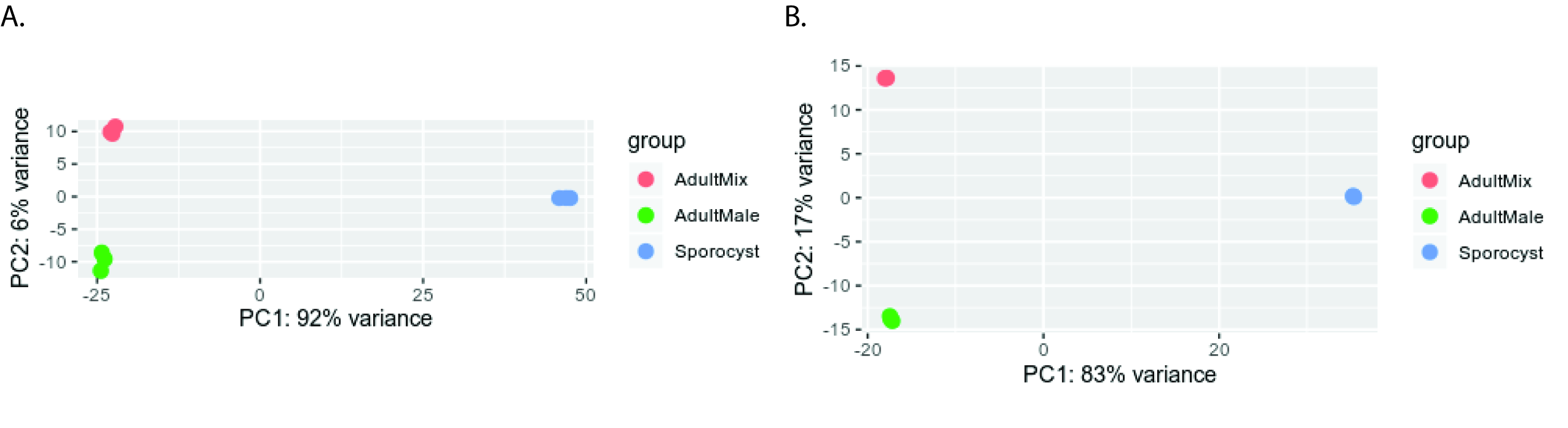

Supplement: Supplemental Material [file KRNB_A_1729594_SM1844.zip › SupFig1_PCA.tif]

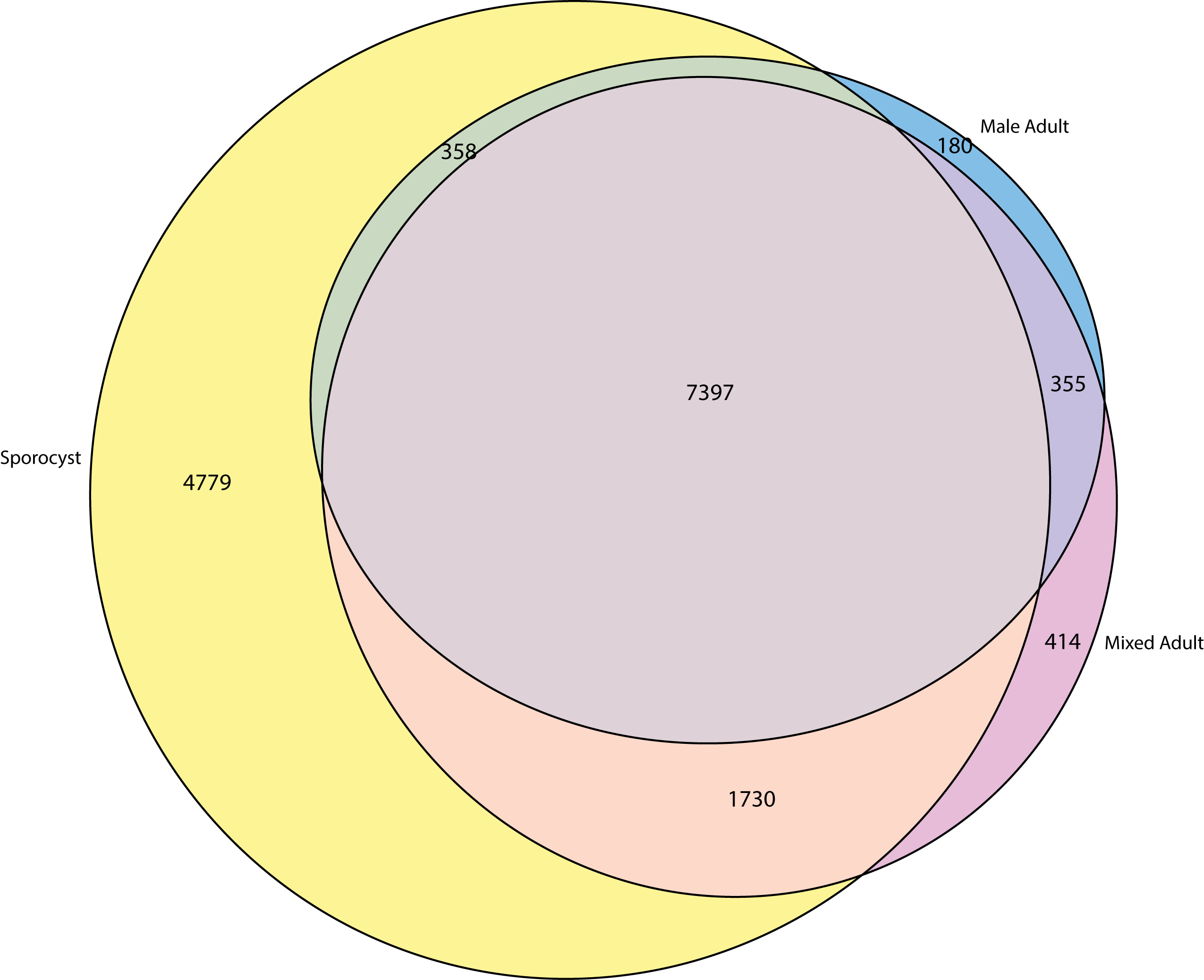

Supplement: Supplemental Material [file KRNB_A_1729594_SM1844.zip › SupFig2_euler_total_lncRNA.tif]

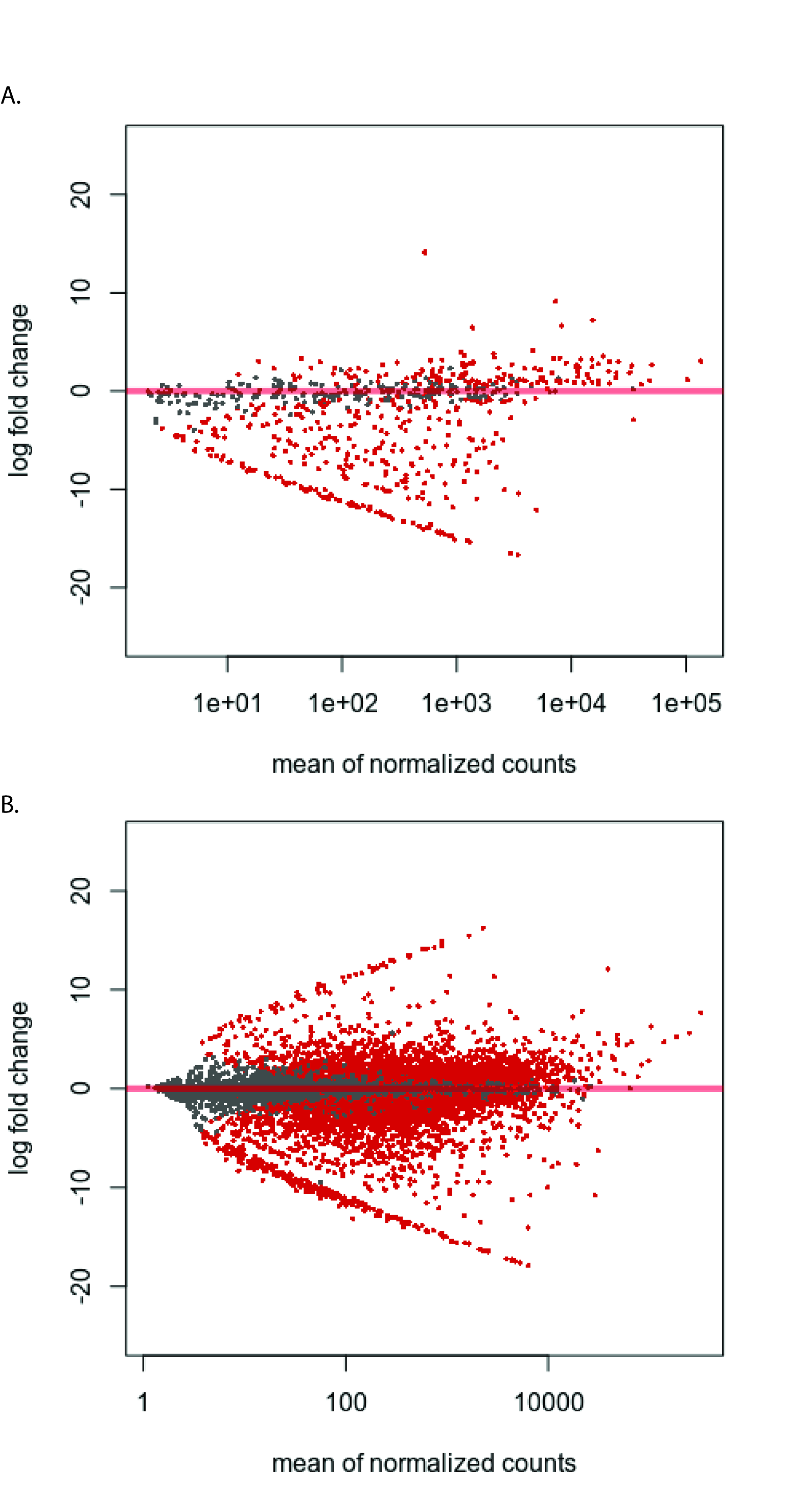

Supplement: Supplemental Material [file KRNB_A_1729594_SM1844.zip › SupFig3_MAplot_Cerc_vs_Spor.tif]

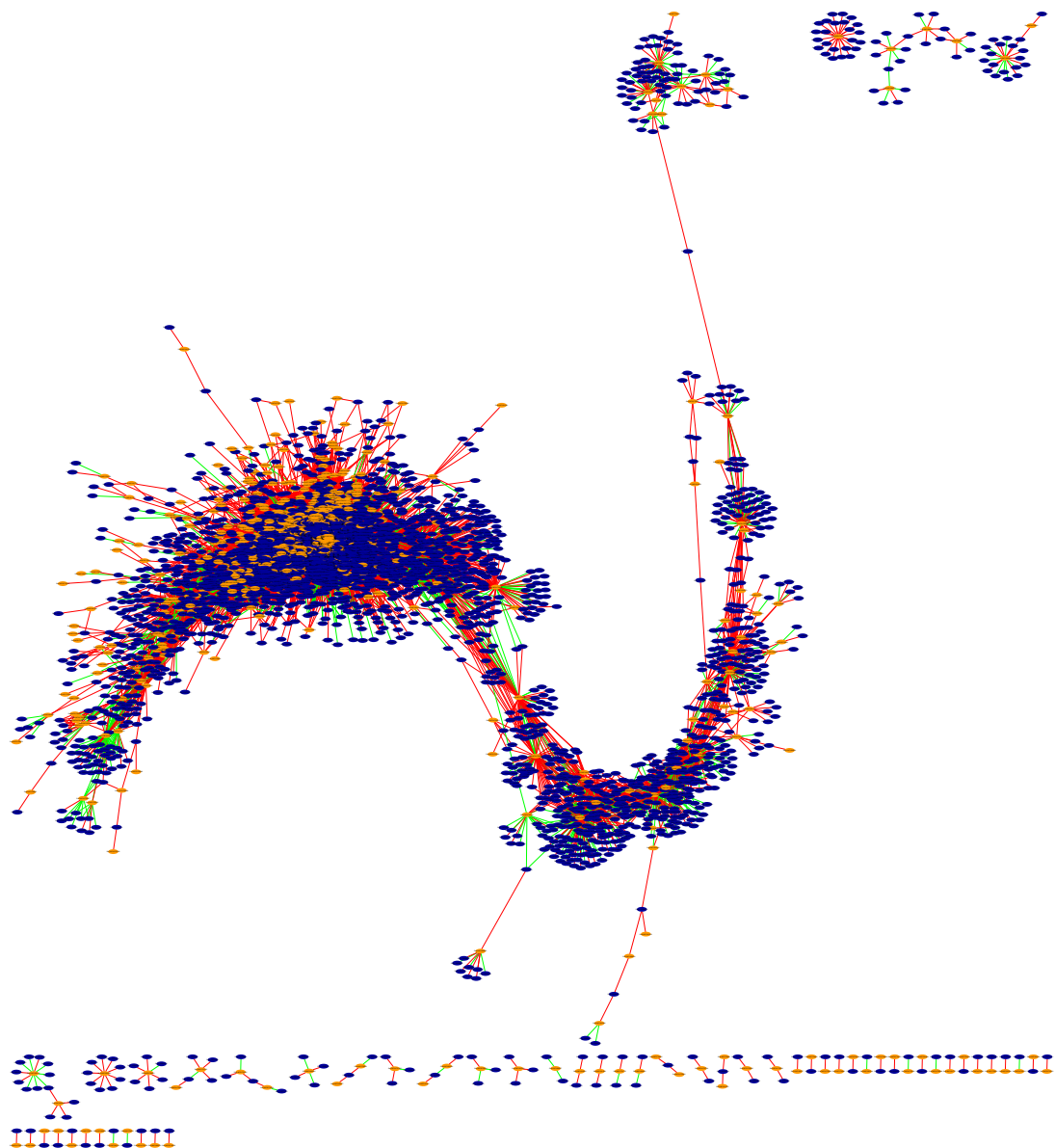

Supplement: Supplemental Material [file KRNB_A_1729594_SM1844.zip › SupFig4A_new_lncRNA_PC.pdf]

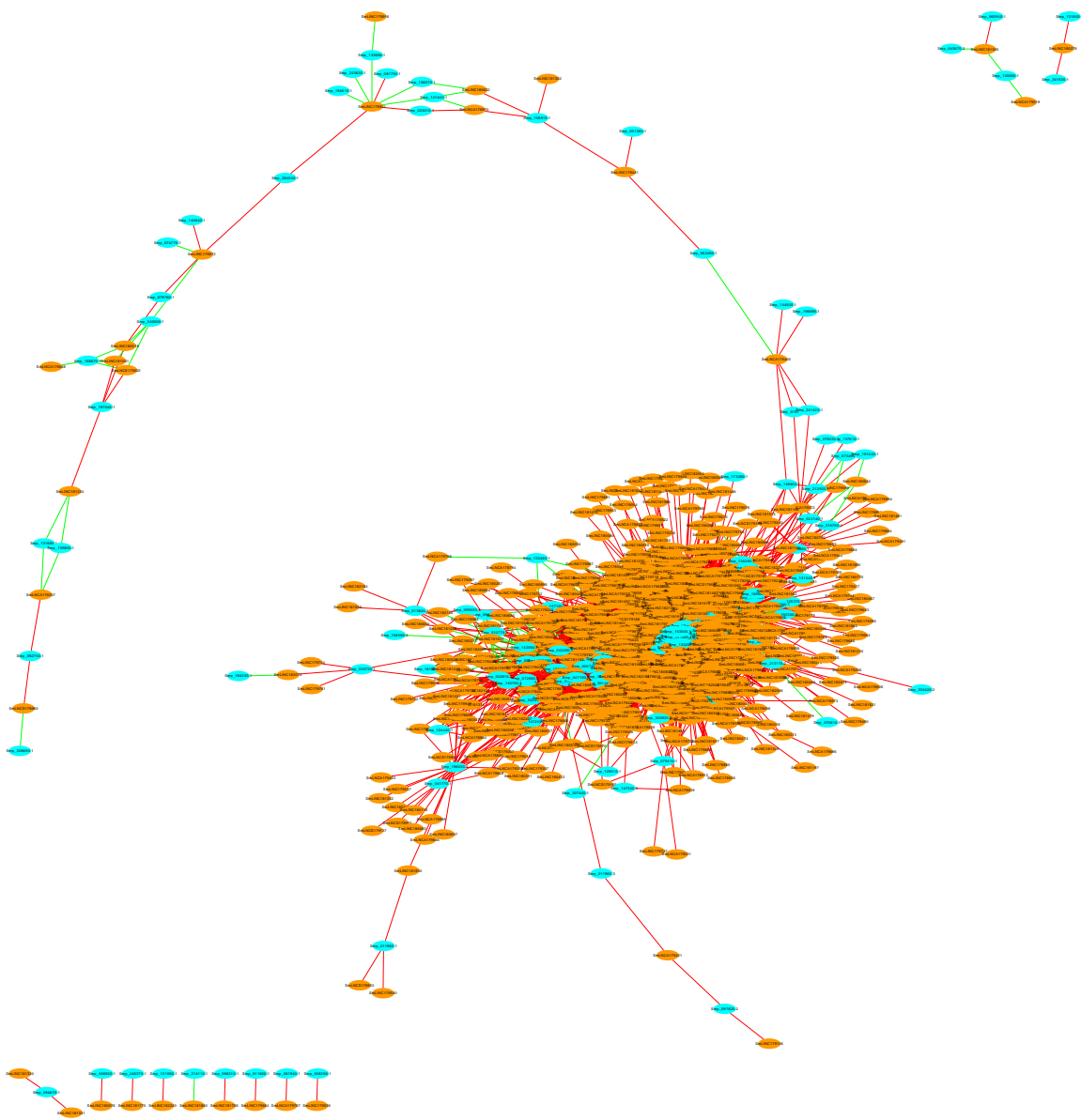

Supplement: Supplemental Material [file KRNB_A_1729594_SM1844.zip › SupFig4B_new_lncRNA_kinase.pdf]

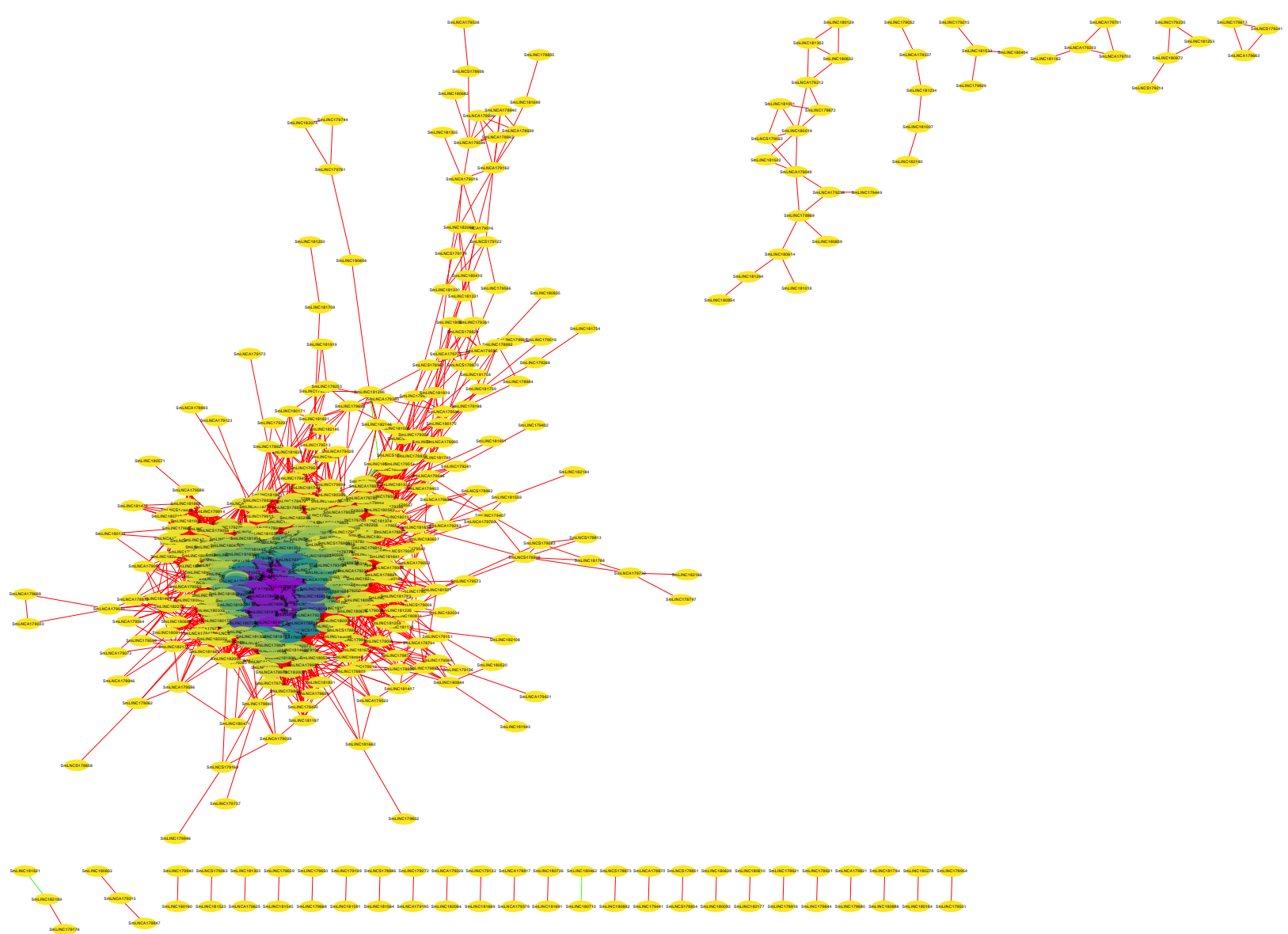

Supplement: Supplemental Material [file KRNB_A_1729594_SM1844.zip › SupFig4C_new_lncRNA_lncRNA.pdf]
